# Harris' Medical and Dental Dictionary—A New Edition

**Published:** 1877-11

**Authors:** 


*Harris' Medical and Dental Dictionary.*—A new and carefully revised edition of the above work is in press, the work being under the supervision of Prof. F. J. S. Gorgas. The constantly changing and improving methods of dentistry, and the rapid onward march of dental and all other science, makes this revision a necessity. Text books can never be quite up with the times—we must look to the journals for this—but it is important that they should be as close up as possible, and in this day of rapid movements it does not take long to be behindhand. The new edition will give us all that is standard, the obsolete eliminated and much that is new and valuable introduced. A more extended notice will be made when the work appears.

